# Experimental Investigation of Nanodiamond Reinforcement in PU for Enhancing Mechanical, Scratch, Rheological, Thermal, and Shape-Memory Properties

**DOI:** 10.3390/polym17212947

**Published:** 2025-11-04

**Authors:** Markapudi Bhanu Prasad, Nashmi H. Alrasheedi, P. S. Rama Sreekanth, Borhen Louhichi, Santosh Kumar Sahu, Nitesh Dhar Badgayan

**Affiliations:** 1 School of Mechanical Engineering, VIT-AP University, Besides A.P. Secretariat, Amaravati 522237, India; 2 Department of Mechanical Engineering, College of Engineering, Imam Mohammad Ibn Saud Islamic University (IMSIU), Riyadh 11432, Saudi Arabia; 3 Engineering Sciences Research Center (ESRC), Deanship of Scientific Research, Imam Mohammad Ibn Saud Islamic University (IMSIU), Riyadh 11432, Saudi Arabia; 4 KPMG, Mumbai 400011, India

**Keywords:** PU, ND, shape memory polymer, mechanical properties, thermal properties, shape memory properties

## Abstract

Shape-memory polymers (SMPs) are a unique class of smart materials capable of recovering their original shape upon external stimuli, with thermoresponsive polyurethane (PU) being one of the most widely studied systems. However, the relatively low mechanical strength, thermal stability, and durability of PU limit its broader functional applications. PU/ND composites containing 0.1–0.5 wt.% ND were fabricated via melt blending and injection molding method. The objective was to evaluate the effect of ND reinforcement on the mechanical, scratch, thermal, rheological, and shape-memory properties. Results show that tensile strength increased up to 114% and Young’s modulus by 11% at 0.5 wt.% ND, while elongation at break decreased due to restricted chain mobility. Hardness improved by 21%, and scratch resistance was significantly enhanced, with the coefficient of friction reduced by 56% at low loads. Thermal stability was improved, with the maximum degradation temperature shifting from 350 °C (pure PU) to 362 °C (0.5 wt.% PU/ND) and char yield increasing by 34%. DSC revealed an increase in glass transition temperature from 65 °C to 68.6 °C. Rheological analysis showed an 89% reduction in damping factor (tan δ), indicating enhanced elasticity. Shape-memory tests confirmed notable improvements in both shape fixity and recovery ratios across successive cycles compared to neat PU, with the highest enhancements observed for the 0.5 wt.% PU/ND nanocomposite—showing up to 7.6% higher fixity and 32% higher recovery than pure PU. These results demonstrate that ND reinforcement effectively strengthens PU while preserving and improving its shape-memory behavior, making the composites promising candidates for high-performance smart materials in sensors, actuators, and aerospace applications.

## 1. Introduction

Polymers are synthetic materials consisting of repeating structural units known as monomers. These materials are widely utilized due to their exceptional physical and chemical properties [1]. Among them, shape-memory polymers (SMPs) are a unique class of smart materials that can recover their original shape after deformation when exposed to specific external stimuli [2]. Such stimuli include heat [3], electric current [4], light [5], magnetic force [6], water [7], microwave radiation [8], mechanical pressure [9], and chemical solvents [10]. Among various SMPs, heat-responsive types have garnered the greatest attention due to their effective and controllable response to temperature variations [11]. Polyurethane (PU), a thermoplastic elastomer, combines the durability of plastics with the flexibility and resilience of rubber. As a thermoresponsive SMP, PU has been extensively studied owing to its excellent shape-memory ability, low density, and ease of processing [12,13]. However, its relatively poor mechanical and thermal properties limit broader applications [14]. To overcome these drawbacks, researchers have investigated the incorporation of nanofillers into the PU matrix, which can significantly enhance its structural and functional performance [15].

Sanaka et al. [16] showed that PU reinforced with MXene (0–1.0 wt.%) exhibited optimum mechanical performance at 0.5 wt.% loading, with tensile modulus, tensile strength, and hardness improving by 22, 281, and 19%, respectively, compared to pure PU. Thermal analysis also revealed increases in melting temperature (T_m_), enthalpy of melting (ΔH_m_), and crystallinity, with maximum improvements of 34% at 0.5 wt.% MXene. Similarly, graphene (Gr)-reinforced PU composites displayed superior mechanical performance, with 0.05 PU/Gr achieving 25%, 26%, and 31% enhancements in Young’s modulus, tensile strength, and flexural strength, respectively [17]. Other nanofillers have also demonstrated significant potential. PU/MWCNT nanocomposites (0–1.0 wt.%) achieved a 25% increase in elastic modulus, a 21% improvement in ultimate tensile strength, and an 11% increase in elongation at break at 1.0 wt.% [18]. Graphene-reinforced PU composites (0–0.7 wt.%) exhibited dramatic improvements, with the modulus of elasticity increasing by 320% and elongation at break by 134% at 0.7 wt.% loading [19]. PU reinforced with graphene nanoplatelets (0.25–0.75 wt.%) showed increases in Young’s modulus by up to 127%, along with improved crystallinity confirmed by DSC analysis, indicating GNPs acted as efficient nucleating agents [20]. PU nanocomposites reinforced with multi-walled carbon nanotubes (MWCNTs) and halloysite nanotubes (HNTs) at 0, 0.5, and 1 wt.% exhibited significant improvements in both tensile and thermal properties. The incorporation of 0.1 wt.% PU/MWCNT yielded the highest tensile strength (23.5 MPa) and glass transition temperature (69 °C) [21]. Similarly, PU reinforced with graphene (GR) at varying weight percentages (PUGR000, PUGR025, PUGR050, and PUGR100) demonstrated enhanced thermal stability, increasing from 343 °C to 400 °C due to the barrier effect of graphene. Mechanical properties also improved, with the tensile modulus and strength increasing by 59% and 12%, respectively, and maximum values (2.98 MPa and 1.76 MPa) were achieved at 20 wt.% GR. All GR-reinforced samples displayed shape recovery around 55 °C, with consistent fixity; however, the recovery ratio decreased slightly with increasing GR content, attributed to increased stiffness [22]. Reinforcement of PU with 0–10 wt.% HNTs also improved its tensile behavior. Compared to virgin PU, the addition of 8 wt.% HNTs increased tensile strength by 30% and tensile modulus by 47% [23]. When a hybrid CNT–HNT filler was incorporated into PU, the modulus, tensile strength, and hardness improved by 69%, 29%, and 1%, respectively, compared to pure PU [24]. A PU composite reinforced with MWCNTs and titanium dioxide (TiO_2_) further exhibited excellent mechanical properties, including a tensile stress of 4.46 MPa, elongation at break of 49%, and a Young’s modulus of 9.17 MPa [25]. Nanodiamond (ND)-reinforced PU micro/nanofiber membranes showed remarkable improvements in performance. Incorporation of 5.5 wt.% NDs enhanced Young’s modulus, elongation at break, and tensile strength, by 29, 66, and 105%, respectively [26]. PU reinforced with 10 wt.% MWCNTs exhibited a 124% increase in elastic modulus and a 53% rise in hardness compared to pure PU [27]. Likewise, thermoplastic PU (TPU) reinforced with HNTs showed significant enhancements, with storage modulus and mechanical modulus increasing by 185% and 122%, respectively, as HNT loading increased [28]. PU filled with 0.5–2 wt.% MWCNTs displayed highly improved shape recovery ratios of 90–100%, in contrast to the lower recovery ability of neat PU [29]. The addition of NDs at 0.1–0.5 wt.% to PU further improved mechanical properties, with tensile strength increasing by 71–113% and flexural strength by 23–97% [30]. Overall, these studies demonstrate the effectiveness of incorporating various nanofillers in enhancing the mechanical, thermal, and shape-memory performance of PU. A comparative overview of the reported PU chemical structures, filler types, evaluated properties, and corresponding outcomes is provided in Table 1. It is noteworthy that while some studies focused on tailoring the PU backbone without introducing fillers, others investigated filler-based reinforcement without specifying the PU chemical structure, collectively offering a broad perspective on structure–property relationships in PU-based systems.

From the literature survey, it is evident that most studies on PU nanocomposites have primarily focused on incorporating fillers such as carbon nanotubes (CNTs), halloysite nanotubes (HNTs), graphene oxide (GO), and nanosilica (NS). Although these fillers have shown notable improvements in PU performance, a significant research gap remains in the development of ND-reinforced PUs. NDs are distinguished by their exceptional hardness, originating from strong carbon–carbon covalent bonds, and an ultrahigh modulus of approximately 1 TPa [44,45]. These properties make NDs promising candidates for enhancing both the mechanical and thermal properties of polymer matrices. In addition, limited attention has been given to understanding how the intrinsic chemical structure of PU influences its interaction with such fillers, particularly in relation to phase separation behavior and shape-memory performance [46]. Variations in the soft and hard segment composition, crosslinking density, and functional group distribution play a crucial role in determining filler–matrix compatibility and stress transfer efficiency. The novelty of the present study lies in the strategic design of ND-reinforced PU nanocomposites, where the interplay between ND loading and PU chemical structure is exploited to enhance mechanical strength, thermal stability, and shape-memory behavior. Unlike conventional PU-based SMPs or composites reinforced with other nanofillers, this system achieves an optimized balance between structural integrity and functional responsiveness, thereby offering new potential for high-performance smart material applications.

## 2. Materials and Methods

### 2.1. Materials

**Figure 1:**
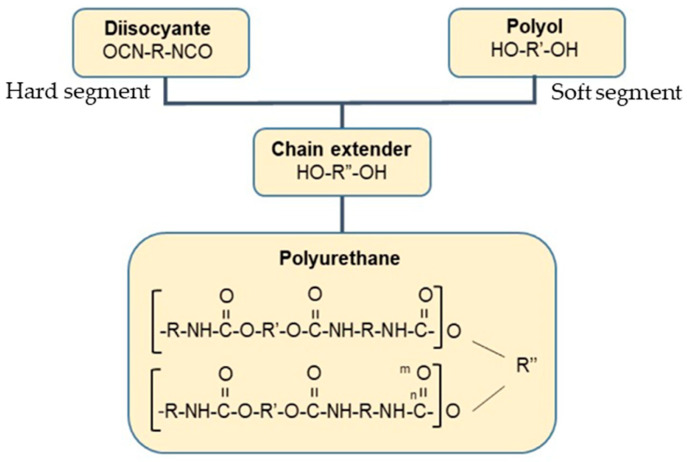
Chemical structure of PU with soft segment (Polyol), hard segment (Diisocyanate, chain extender) and alternating soft and hard segments [43].

**Figure 2:**
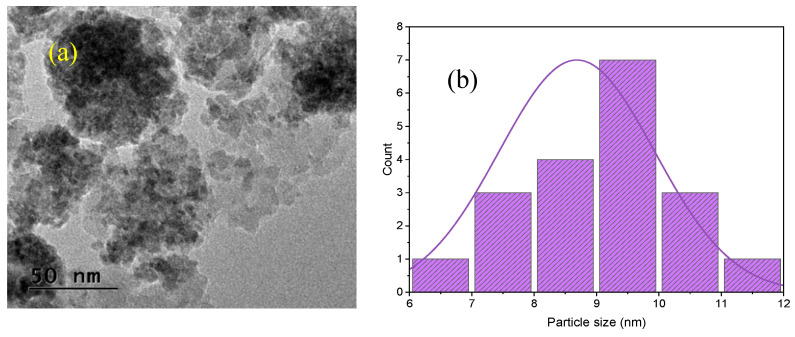

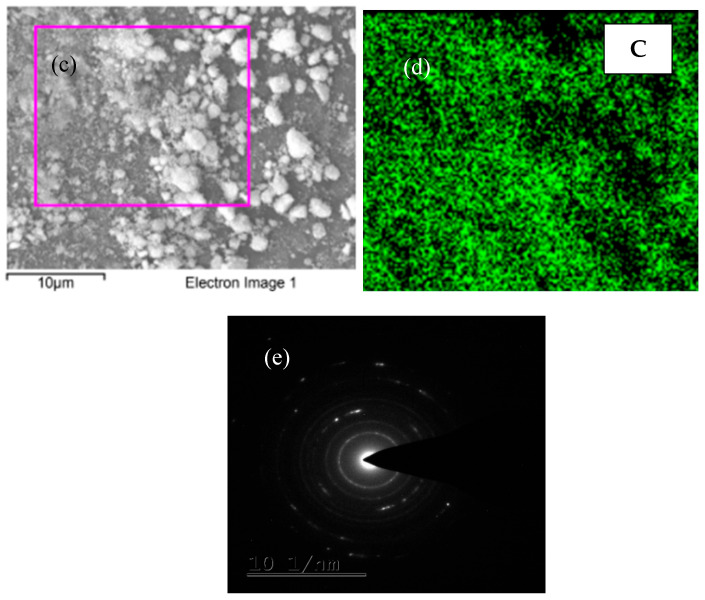
(**a**) TEM image of ND; (**b**) Particle size distribution of ND; EDX mapping at (**c**) Area selected (**d**) Elemental mapping of carbon (C); (**e**) SAED pattern of ND.

PU granules were procured from SMP Technologies Inc., Tokyo, Japan, while ND fillers were obtained from Nano Research Element Inc., Delhi, India. The PU pellets had a diameter of 7–8 mm, a density of approximately 0.834 g/cm^3^, and a glass transition temperature (T_g_) around 65 °C. The chemical structures of the PU components are illustrated in Figure 1 [43]. As shown in Figure 1 the soft segment consists of a polycaprolactone (PCL) polyol, while the hard segment is diisocyanate (MDI) with chain extender 1,4-butanediol. The alternating soft and hard segments is shown at the end of flow chart, where the flexible PCL chains constitute the soft domains, and the MDI–butanediol linkages form the rigid hard domains responsible for mechanical strength and thermal stability. The ND fillers were supplied in powder form, with average particle size below 10 nm, a density of 3.18 g/cm^3^, a purity greater than 99%, and a specific surface area of 350 m^2^/g. Transmission Electron Microscopy (TEM) was employed to examine the morphology of the as-received NDs. As shown in Figure 2a, the NDs exhibit a dot-like morphology. The particle size distribution (Figure 2b) indicates an average size of less than 10 nm. Energy Dispersive X-ray (EDX) mapping (Figure 2c,d) confirms the presence of carbon, while the Selected Area Electron Diffraction (SAED) pattern (Figure 2e) reveals distinct crystalline rings.

### 2.2. Fabrication of Composite Samples

**Figure 3:**
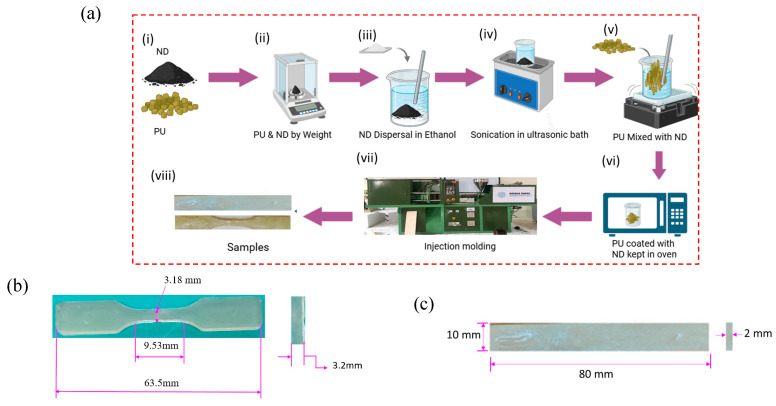
(**a**) Schematic for fabrication steps of composite samples; fabricated sample with dimension: (**b**) tensile; (**c**) bending test.

The composite samples were prepared using a PU matrix reinforced with ND particles at weight percentages of 0.1, 0.2, 0.3, and 0.5 wt.%. The overall fabrication process is illustrated in Figure 3a. First, the required amount of ND nanoparticles was chemically modified following the procedure reported in the literature [45]. In this stage, the as received NDs underwent oxidation using a mixed acid solution composed of concentrated sulfuric acid (98%) and nitric acid (70%) in a 3:1 volume ratio. The blend was first sonicated in an ultrasonic bath (Branson 2510; OPTO-LAB, Modena, Italy) and then transferred to hot water maintained at 90 °C, where it was continuously stirred for 10 h. Afterward, the suspension was diluted with distilled water, filtered, and repeatedly washed to eliminate any remaining acid traces. The collected material was dried in an oven at 80 °C for 4 h. This oxidation process introduced oxygen-containing functional groups, mainly carboxyl groups, onto the ND surface, forming carboxylated ND (ND–(COOH)_n_). These groups improve the NDs dispersion and promote stronger interfacial bonding with the PU matrix. The modified ND nanoparticles were accurately weighed using an electronic balance and dispersed in ethanol at a ratio of approximately 1:0.5. The resulting nanofluid was continuously stirred and sonicated to achieve a uniform dispersion. Subsequently, the required amount of PU pellets was added to the prepared nanofluid in a beaker and stirred using a glass rod while placed on a hot plate. To eliminate residual moisture, the mixture was oven-dried for 24 h. After complete drying, the PU coated with ND nanofillers was transferred to an injection molding machine fitted with ASTM-standard dies for the fabrication of tensile and flexural test specimens. Composite samples were successfully produced with ND loadings of 0.1, 0.2, 0.3, and 0.5 wt.% PU/ND, along with pure PU samples prepared for comparison. The fabricated tensile and bending test specimens were prepared according to ASTM D638 type V [47] and ISO 178 standards [48], and their detailed dimensions are shown in Figure 3b,c, respectively.

### 2.3. XRD Test

The presence of crystalline peaks in the PU and PU/ND composites were examined using X-ray diffraction (XRD). The XRD analysis was performed on a Miniflex600-C (Rigaku, Tokyo, Japan) diffractometer equipped with Cu Kα radiation (λ = 1.54 Å) over a scanning range of 10–90°. Five repetitions of each sample were conducted, and the average values were reported.

### 2.4. Tensile Test

Tensile testing was carried out using a H10KL (Tinius Olsen India Pvt. Ltd., Uttar Pradesh, India) universal testing machine (UTM) in accordance with ASTM D638 standards [47], under ambient conditions and at a cross head displacement rate of 2 mm/min. For each composition, six individual specimens were tested under identical conditions, and the mean values were calculated and reported. Separate specimens were used for each tensile test to eliminate any effect of prior plastic deformation. To further analyze the fracture behavior, the fractured surfaces of PU composites after tensile testing were examined using a Scanning Electron Microscope (SEM). Before imaging, all samples were gold-sputtered to ensure conductivity. SEM observations were carried out on a ZEISS EVO10 (Zeiss, Oberkochen, Germany) system operated at an accelerating voltage of 5–15 kV. The analysis focused on the fracture regions to evaluate surface morphology, filler dispersion, and the interfacial integration of ND particles within the PU matrix.

### 2.5. Scratch and Hardness Test

Scratch testing of the PU composites was performed using a TR-101-IAS (DUCOM, Tokyo, Japan) scratch tester equipped with a diamond-tipped indenter. Tests were conducted under normal loads of 20 N, 40 N, and 60 N, with the indenter traversing a distance of 10 mm across the specimen surface. Hardness measurements were carried out using a Vickers hardness tester (model FMV1-MC-AT, R.S.Scientific, Kolkata, West Bengal). A diamond indenter was employed to create indentations on the specimen surface under an applied load of 1 kg with a dwell time of 20 s. The Vickers hardness number (HV) was calculated using Equation (1) [49]:

(1) $$HV=1.889\frac{F}{d^{2}}$$

where *F* is the applied load in newtons (N), and *d* is the diagonal length of the square indentation in millimeters (mm). For both scratch and hardness testing, six repetitions were performed for each sample, and the average values were recorded.

### 2.6. DSC and TGA Test

The thermal behavior of the composites was evaluated using an STA 8000 (PerkinElmer Inc., Waltham, MA, USA), which simultaneously performed Differential Scanning Calorimetry (DSC) and Thermogravimetric Analysis (TGA) within the temperature range of 25–600 °C at a constant heating rate of 10 °C/min. A continuous nitrogen flow of 20 mL/min was maintained throughout the tests to purge residual gases. For each measurement, approximately 12 mg of PU composite sample was used. All tests were repeated five times, and the average values were recorded.

### 2.7. Rheological Test

The viscoelastic behavior of the samples was evaluated using a rheometer (MCR 102, Anton Paar, Graz, Austria) in oscillatory mode. Tests were performed in frequency sweep mode using a parallel plate configuration (40 mm diameter) with a strain amplitude of 0.05 over a frequency range of 0.1–100 rad/s. During the measurements, the storage modulus (G′), loss modulus (G″), and damping factor (tan δ) were recorded as functions of frequency. This procedure represents a dynamic rheological test, which is distinct from dynamic mechanical analysis (DMA).

### 2.8. Heat-Responsive Shape-Memory Test

**Figure 4:**
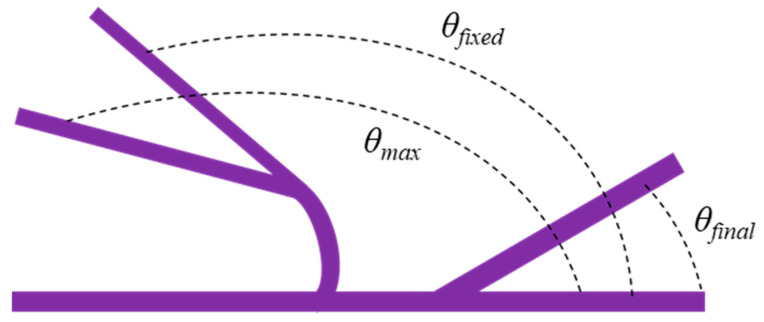
Schematic illustration of angle measurement during the recovery process of rectangular sample.

The heat-responsive shape-memory behavior of the rectangular specimens (80 × 10 × 2 mm) was evaluated using a bending-over-cylinder (fold–deploy) method, as illustrated in Figure 4 [50,51]. The procedure involved the following steps: (i) the specimens were heated to 80 °C, which is above the glass transition temperature (T_g_) of the PU matrix, and maintained for 5 min to soften the material; (ii) the softened specimens were bent over a cylindrical rod to achieve the target maximum deformation angle (θ_max_) and held on the mandrel for 15 min to ensure uniform curvature and complete deformation; (iii) the deformed specimens were immediately quenched in room-temperature water while constrained to fix the temporary shape, and the fixed bending angle (θ_fixed_) was recorded; (iv) for the recovery test, the specimens were placed in a water bath maintained at 80 °C, and the shape recovery process was photographed at 10 s intervals for a total duration of 60 s to monitor the recovery behavior by measuring the recovery angle (θ_final_). Each specimen underwent three consecutive shape-memory cycles to evaluate repeatability and stability. The shape fixity ratio (R_f_) and shape recovery ratio (R_r_) were calculated using Equations (2) and (3), following the methodology reported in [51].

(2) $$R_{f}=\frac{\theta_{fixed}}{\theta_{max}}\times 100\%$$

(3) $$R_{r}=\frac{\theta_{fixed}-\theta_{final}}{\theta_{fixed}}\times 100\%$$

## 3. Results and Discussion

### 3.1. XRD Results

**Figure 5:**
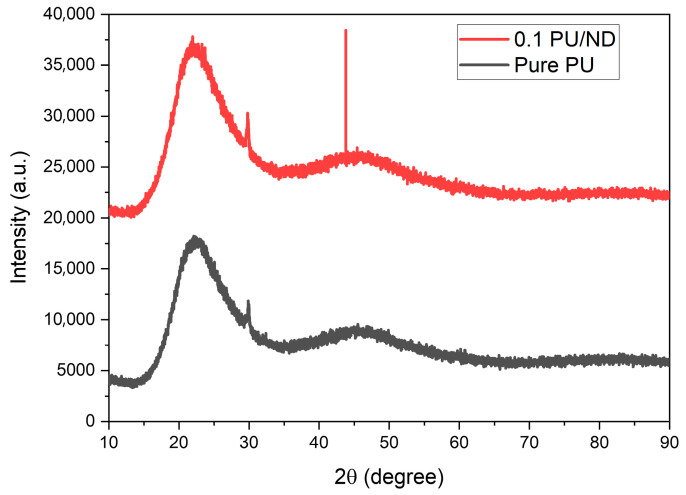
XRD results of pure PU and 0.1 PU/ND.

Figure 5 presents the X-ray diffraction (XRD) patterns of pure PU and PU reinforced with 0.1 wt.% ND over the 2θ range of 10–90°. The pure PU sample exhibits a broad diffraction halo centered around 20–25° (2θ), which is characteristic of its semi-crystalline polymer. In addition, a weak reflection is observed near 26° (2θ), which can be attributed to the partial ordering of hard-segment microdomains. Upon the incorporation of NDs, the 0.1 PU/ND nanocomposite displays additional crystalline features, most notably a distinct peak at 43.8° (2θ) that corresponds to the (111) plane of cubic diamond [43]. The appearance of this diamond-specific reflection, although weak due to the low filler concentration, provides clear evidence for the presence and successful dispersion of ND crystallites within the PU matrix.

### 3.2. Tensile Properties

**Figure 6:**
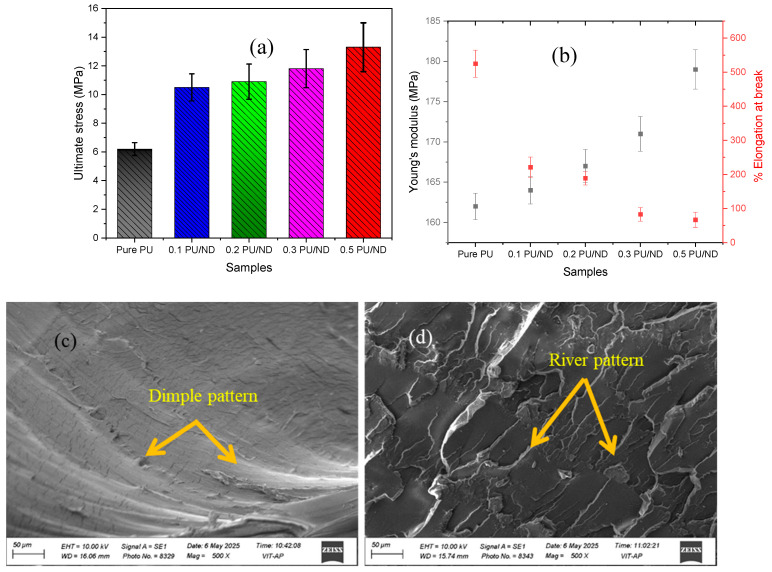
(**a**) Ultimate tensile strength; (**b**) Young’s modulus and % elongation at fracture; SEM fractography of (**c**) pure PU and (**d**) 0.5 PU/ND.

The ultimate tensile stress, Young’s modulus, and percentage elongation (at fracture) were obtained from the stress–strain curves for PU and PU/ND composites [30]. Figure 6a presents the ultimate tensile stress values for different PU/ND compositions. The pure PU exhibited the lowest ultimate stress of 6.2 ± 0.5 MPa, while the incorporation of ND progressively enhanced the tensile strength. The ultimate stress increased by approximately 69%, 78%, 95%, and 114% for 0.1 PU/ND, 0.2 PU/ND, 0.3 PU/ND, and 0.5 PU/ND, respectively, compared to pure PU. Figure 6b depicts the trend of Young’s modulus and percentage elongation at break for various composite samples. The Young’s modulus of neat PU was measured as 162 ± 2 MPa. With the addition of ND, the modulus showed a steady increase, reaching values enhanced by 1%, 3%, 5%, and 11% for 0.1, 0.2, 0.3, and 0.5 PU/ND, respectively. In contrast, the percentage elongation at break displayed a decreasing trend with increasing ND content. The virgin PU exhibited an elongation of about 525% ± 40, which decreased to 221%, 189%, 83%, and 67% for 0.1, 0.2, 0.3, and 0.5 PU/ND, respectively. The increase in ultimate strength and Young’s modulus can be attributed to the exceptionally high stiffness of ND of (1 TPa) [44], which enables ND to act as effective reinforcing fillers within the PU matrix. The uniform dispersion of ND particles, combined with strong interfacial bonding between PU and ND, promotes efficient stress transfer from the flexible polymer chains to the rigid nanofillers, thereby enhancing overall mechanical performance [52]. In contrast, the decrease in elongation at break arises from restricted chain mobility, as the rigid NDs hinder segmental motion of the PU matrix. This interpretation is supported by SEM fractography (Figure 6c,d): pure PU exhibited a dimpled pattern surface characteristic of ductile failure (Figure 6c), whereas the 0.5 wt.% PU/ND nanocomposite displayed rough, river-like pattern fracture features indicative of brittle behavior (Figure 6d).

### 3.3. Scratch Test Results

**Figure 7:**
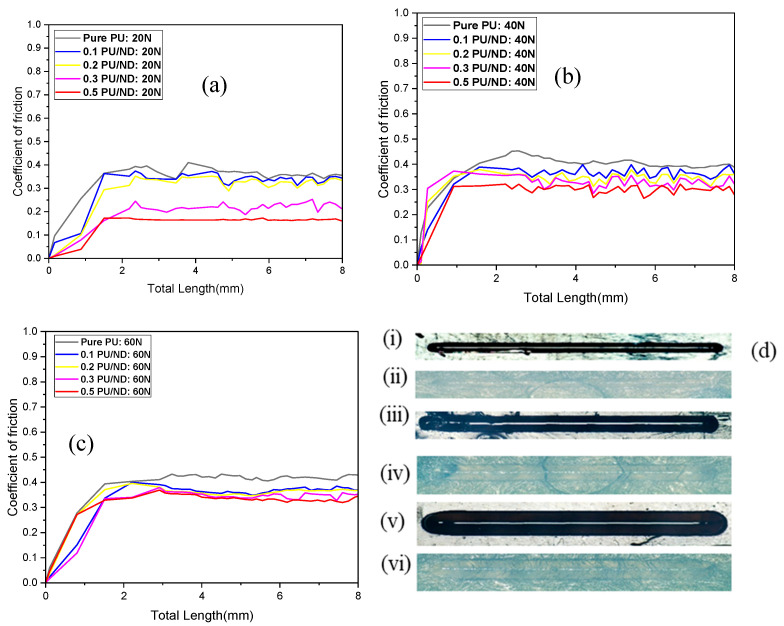
COF vs. total Length at (**a**) 20 N; (**b**) 40 N; and (**c**) 60 N; (**d**) scratch images for pure PU sample at (**i**) 20 N; (**iii**) 40 N; (**v**) 60 N; and 0.5 PU/ND sample at (**ii**) 20 N; (**iv**) 40 N; (**vi**) 60 N load.

Scratch testing was conducted to assess the scratch resistance and frictional response of polymer composites under different applied loads. Figure 7a–c presents the average coefficient of friction (COF) for pure PU and PU/ND nanocomposites at 20 N, 40 N, and 60 N. As shown in Figure 7a, at 20 N, pure PU exhibits an average COF of 0.34 ± 0.02 and produces deeper, more pronounced grooves in the scratch images, indicating limited resistance to surface deformation. Incorporation of NDs progressively reduces the average COF to 0.32 (0.1 wt.%), 0.30 (0.2 wt.%), 0.20 (0.3 wt.%), and 0.15 (0.5 wt.%), reflecting enhanced scratch performance. As the load increases to 40 N, shown in Figure 7b, the average COF of pure PU rises to 0.38 ± 0.03, and scratch images reveal wider and deeper grooves caused by increased adhesion and ploughing under higher contact pressure. In contrast, PU/ND composites retain significantly lower average COF values (0.34, 0.33, 0.31, and 0.28) ± 0.03 with narrower and shallower grooves, demonstrating improved scratch resistance. At 60 N as shown in Figure 7c, the average COF of pure PU increases further to 0.39 ± 0.02, and the wear tracks show severe material damage with deep, distorted grooves, indicating unstable sliding despite a slight friction reduction from transfer film formation. PU/ND composites, however, maintain lower average COF values (0.35, 0.34, 0.33, and 0.32) ± 0.03. This behavior confirms that NDs incorporation strengthens the PU matrix, restricts plastic deformation, and facilitates the formation of a stable third-body layer that reduces COF.

Figure 7d highlights these effects, showing wear tracks for pure PU and 0.5 PU/ND at 20 N (i,ii), 40 N (iii,iv), and 60 N (v,vi). Across all loads, PU/ND composites exhibit smoother and less pronounced grooves compared to pure PU, with the improvement being more pronounced at higher ND content. The higher COF for pure PU is attributed to its low hardness and high plastic deformation, which intensify adhesion and ploughing under load. In contrast, the lower COF and reduced groove depth in PU/ND composites result from increased hardness, enhanced load-bearing capacity, and the lubricating effect of NDs forming a protective interfacial layer [53]. It is also noted that, at higher loads, the influence of surface morphology and minor compositional variations becomes less dominant compared to the overall material resistance.

### 3.4. Hardness Results

**Figure 8:**
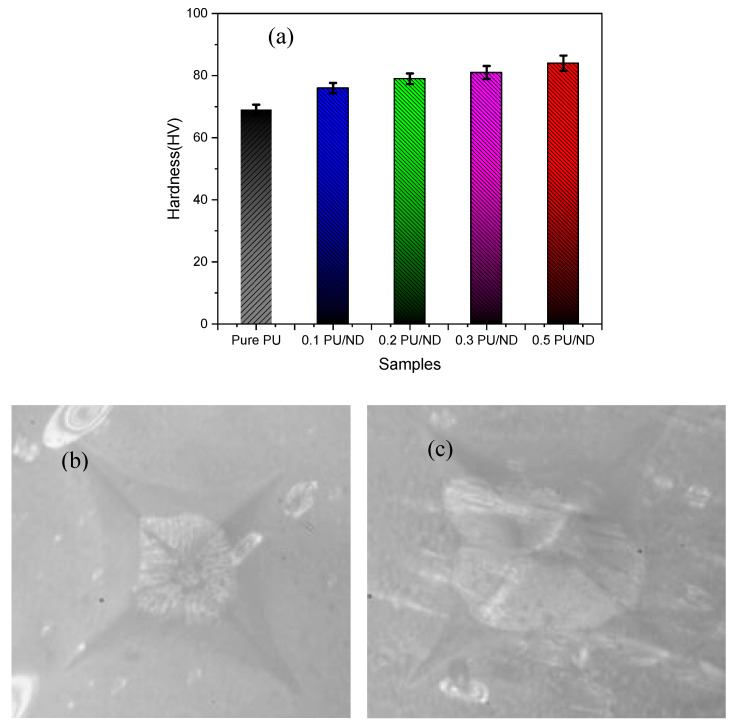
(**a**) Hardness vs. samples; indentation image of (**b**) pure PU and (**c**) 0.5 PU/ND.

The hardness results for all the samples are shown in Figure 8a, and is noted that pure PU exhibits the lowest hardness value of 69 ± 1.6 MPa, reflecting its inherently soft and ductile nature. With the incorporation of ND, the hardness shows a progressive increase of hardness i.e., 10, 14, 17, and 21% for 0.1, 0.2, 0.3, and 0.5 wt.% PU/ND nanocomposites, respectively. This steady enhancement can be attributed to the high intrinsic stiffness of ND particles, which act as reinforcing fillers within the polymer matrix. The strong interfacial interaction between PU chains and ND allows efficient stress transfer, restricting localized deformation under applied load and thereby improving the overall surface hardness. The corresponding indentation images further validate this observation (Figure 8b,c).

As shown in Figure 8b, the indentation marks for pure PU appear smooth and well-defined, consistent with its soft and highly ductile nature. In contrast, as shown in Figure 8c, the 0.5 wt.% PU/ND composite depicts sharper and rougher indentation edges around the impression. This indicates that the addition of ND enhances hardness through reinforcement but also reduces ductility, leading to a comparatively brittle response at higher filler loading [54].

### 3.5. TGA Results

**Figure 9:**
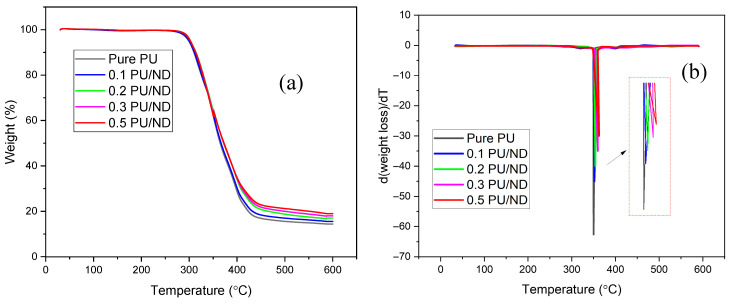
(**a**) TGA vs. temperature; (**b**) DTG vs. temperature.

The thermal stability of neat PU and PU/ND nanocomposites was evaluated using thermogravimetric analysis (TGA) and derivative thermogravimetry (DTG) to probe degradation behavior and the effect of ND reinforcement. The TGA curves (Figure 9a) show an initial mass loss between 50 and 100 °C, attributable to evaporation of physically adsorbed moisture and any residual low-molecular-weight volatiles [55]. The onset temperature (T_onset_), here defined as the temperature at which 2 wt.% mass loss occurs, increases progressively with ND content: T_onset_ is 272 ± 15 °C for pure PU and rises to 273, 275, 280, and 285 ± 16 °C for the 0.1, 0.2, 0.3, and 0.5 wt.% PU/ND nanocomposites, respectively. This systematic increase in thermal stability arises from well-dispersed ND particles that enhance interfacial interactions with PU chains and create a tortuous path for degradation products, thereby restricting chain mobility and delaying decomposition [56]. The residual char observed at 600 °C ± 30 °C increases from 14.1% in pure PU to 15.2%, 16.5%, 17.3%, and 18.9% for the 0.1, 0.2, 0.3, and 0.5 wt.% PU/ND nanocomposites, respectively. This progressive rise in char yield indicates that ND promotes the formation of a stable carbonaceous layer, which acts as a thermal shield and slows further degradation. The DTG curves in Figure 9b reveal a single dominant degradation peak between 350 ± 25 °C and 362 ± 22 °C. For pure PU, the maximum weight-loss rate occurs at ~350 °C ± 25 °C. With ND incorporation, this peak shifts gradually to higher temperatures (352, 354, 359, and 362 ± 24 °C) for 0.1, 0.2, 0.3, and 0.5 wt.% PU/ND, respectively), confirming enhanced thermal stability. The decrease in DTG peak intensity with increasing ND content further suggests a reduction in degradation kinetics due to restricted chain mobility [57].

### 3.6. DSC Results

**Figure 10:**
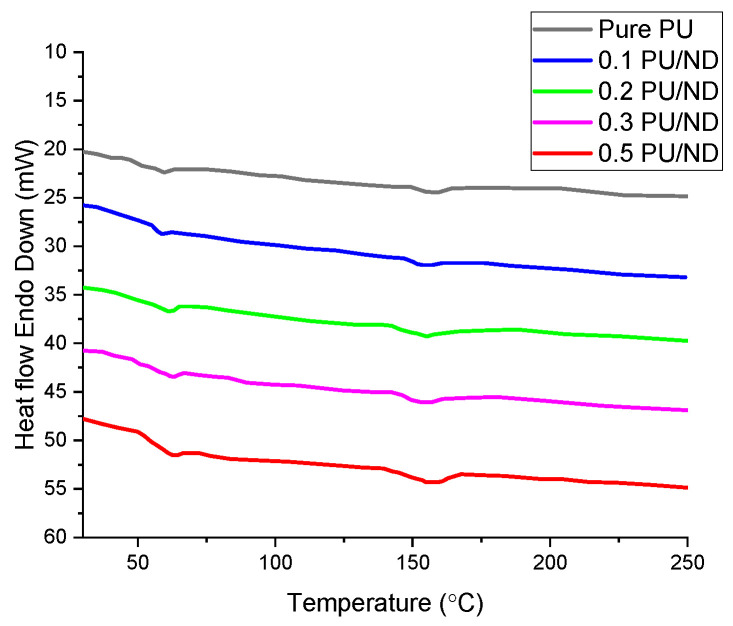
DSC thermograms.

The thermal transitions of PU and PU/ND nanocomposites were examined using differential scanning calorimetry (DSC), and the thermograms are shown in Figure 10. The glass transition temperature (T_g_) of pure PU is observed at 65 ± 4 °C. With ND incorporation, T_g_ gradually increases to 65.5, 67.1, 68.2, and 68.6 ± 5 °C for 0.1, 0.2, 0.3, and 0.5 wt.% PU/ND, respectively. This upward trend reflects the restriction of polymer chain segmental motion due to strong interfacial interactions between PU and ND, which enhance matrix rigidity [58]. The melting temperature (Tm) also shows a slight increase, rising from 161 ± 2 °C in pure PU to 163, 165, 167, and 168 ± 2 °C for 0.1, 0.2, 0.3, and 0.5 wt.% PU/ND, respectively, indicating improved thermal ordering. At the molecular level, the stabilization of the temporary shape is achieved through physical crosslinks formed by interactions between hard segments of PU and the dispersed NDs, revealed in the DSC analysis.

### 3.7. Rheological Properties

**Figure 11:**
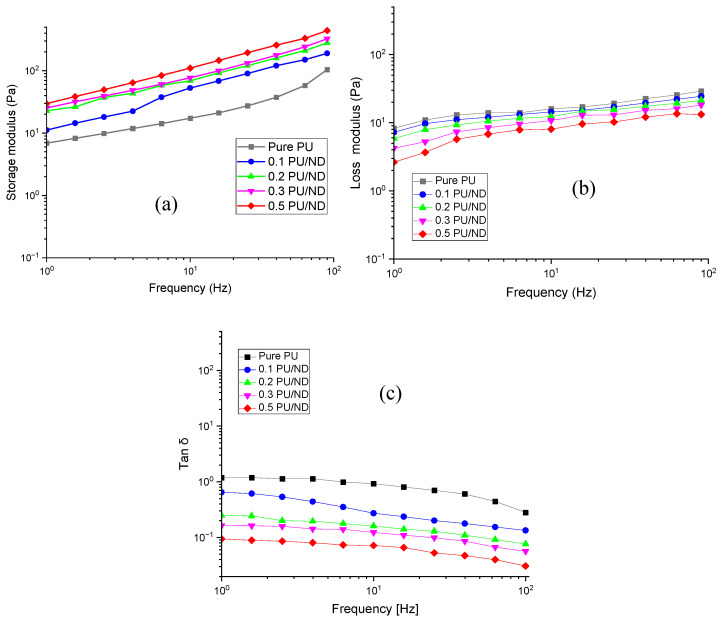
(**a**) Storage modulus vs. frequency; (**b**) loss modulus vs. frequency; (**c**) tan δ vs. frequency.

Rheological analysis was conducted to assess the viscoelastic properties of PU and PU/ND nanocomposites, and the results are presented in logarithmic scale graphs for better visualization of frequency-dependent trends. Figure 11a shows the storage modulus (G′) versus frequency, where all samples exhibit a rising trend due to restricted chain relaxation at higher frequencies. The maximum G′ of 440.05 ± 25 Pa is recorded for 0.5 wt.% PU/ND, with reductions of 26%, 37%, 56%, and 78% for 0.3, 0.2, 0.1 PU/ND, and pure PU, respectively. This demonstrates that the addition of NDs significantly enhances stiffness by constraining the mobility of polymer chains and strengthening filler–matrix interactions [59]. Figure 11b illustrates the loss modulus (G′′), which also increases with frequency, indicating greater energy dissipation at higher oscillation rates. Pure PU shows the highest G′′ of 29.18 ± 2 Pa, which decreases by 16%, 27%, 37%, and 55% upon addition of 0.1, 0.2, 0.3, and 0.5 wt.% ND, respectively. The decline suggests that NDs suppress viscous energy losses by restricting segmental relaxation, shifting the overall response toward a more elastic character [60]. Figure 11c presents the loss factor (tan δ), where pure PU records the highest value of 0.28 ± 0.2, confirming its viscous dominance. With increasing ND loading, tan δ reduces sharply—by ~53%, 75%, 82%, and 89% for 0.1, 0.2, 0.3, and 0.5 wt.% PU/ND, respectively—indicating that higher filler concentrations markedly restrict molecular motion, decrease damping, and enhance the elastic dominance of the nanocomposites [60].

### 3.8. Shape-Memory Behavior

**Figure 12:**
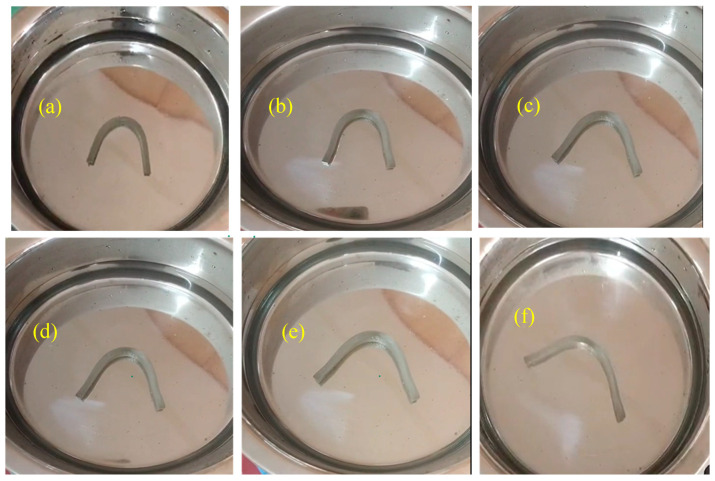
Sequential images showing the shape recovery process of pure PU at cycle 1, recorded at 10 s intervals: (**a**) 10 s, (**b**) 20 s, (**c**) 30 s, (**d**) 40, (**e**) 50 s, and (**f**) 60 s.

**Figure 13:**
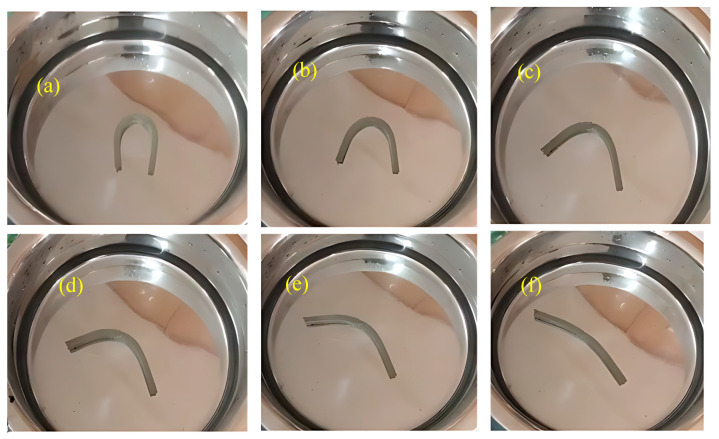
Sequential images showing the shape recovery process of 0.5 PU/ND at cycle 1, recorded at 10 s intervals: (**a**) 10 s, (**b**) 20 s, (**c**) 30 s, (**d**) 40, (**e**) 50 s, and (**f**) 60 s.

**Figure 14:**
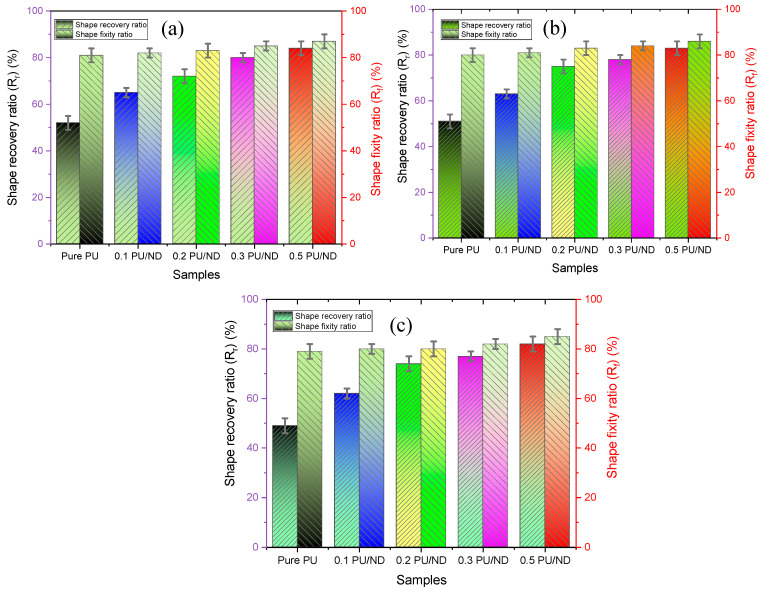
(**a**) Shape recovery vs. shape fixity ratio at cycle 1; (**b**) shape recovery vs. shape fixity ratio at cycle 2; (**c**) shape recovery vs. shape fixity ratio at cycle 3.

The shape-memory performance of PU and PU/ND nanocomposites was evaluated over three successive thermomechanical cycles at 80 °C to assess their ability to fix a temporary shape and subsequently recover their original configuration. Figure 12 and Figure 13 illustrate the deformation behavior of pure PU and the 0.5 wt.% PU/ND nanocomposite during cycle 1 at 10, 20, 30, 40, 50, and 60 s. The corresponding shape fixity and shape recovery ratios for three consecutive cycles, calculated as described in Section 2.8, are presented in Figure 14a–c, with detailed numerical values summarized in Table 2.

For shape fixity, pure PU exhibited values of 81, 80, and 79 ± 3% for cycles 1, 2, and 3, respectively, indicating a slight reduction upon repeated cycling due to minor chain slippage [61]. Incorporation of NDs notably enhanced shape fixity: for 0.1 wt.% PU/ND, the fixity ratios were 82%, 81%, and 80 ± 2%; for 0.2 wt.% PU/ND, 83, 83, and 80 ± 2%; for 0.3 wt.% PU/ND, 85, 84, and 82 ± 2%; and for 0.5 wt.% PU/ND, 87, 86, and 85 ± 3%. The progressive improvement with ND loading demonstrates that ND incorporation promotes more effective physical crosslinking between the hard segments of PU, which act as physical junctions, and the soft segments, which impart flexibility. These enhanced hard–soft segment interactions restrict polymer chain mobility during the programming stage, thereby improving shape retention across multiple cycles. For shape recovery, pure PU displayed recovery ratios of 52, 51, and 49 ± 3% for cycles 1–3, showing a consistent decline with cycling due to irreversible chain relaxation and associated plastic deformation, both of which contribute to permanent structural rearrangements. The addition of NDs resulted in significant improvements: 0.1 wt.% PU/ND exhibited 65, 63, and 62 ± 2%; 0.2 wt.% PU/ND, 72, 75, and 74 ± 3%; 0.3 wt.% PU/ND, 80, 78, and 77 ± 2%; and 0.5 wt.% PU/ND, 84, 83, and 82 ± 3%. These enhancements can be attributed to the strong interfacial interactions and uniform ND dispersion within the PU matrix, which provide additional physical crosslinking sites, stabilize the temporary shape, and facilitate elastic recovery upon heating [62].

## 4. Conclusions

In this study, pure PU and PU/ND nanocomposites with 0.1, 0.2, 0.3, and 0.5 wt.% ND were successfully fabricated via melt blending and injection molding and systematically evaluated for mechanical, scratch, thermal, rheological, and shape-memory properties. ND was found to be an effective nanofiller, significantly enhancing overall performance while retaining the thermoresponsive behavior of PU. Key findings include:

- Tensile strength increased by 114%, Young’s modulus by 11%, and hardness by 21%, while elongation at break decreased due to restricted chain mobility for 0.5 wt.% PU/ND.
- Coefficient of friction reduced by 56%, with shallower grooves observed for 0.5 wt.% PU/ND.
- Maximum degradation temperature increased from 350 °C to 362 °C, and char yield improved by 34% for 0.5 wt.% PU/ND.
- DSC results revealed that T_g_ rose from 65 °C to 68.6 °C, and Tm slightly increased from 161 to 168 ± 2 °C, indicating enhanced chain rigidity and ordering for 0.5 wt.% PU/ND.
- Rheological tests showed a decrease in damping factor (tan δ) by 89% for 0.5 wt.% PU/ND, reflecting improved elasticity.
- Shape-memory performance improved notably, with higher shape fixity and recovery ratios for all PU/ND composites, and the 0.5 wt.% ND sample showing the best overall enhancement due to optimal filler–matrix interactions and physical crosslinking.

Compared to other PU-based nanocomposites reported in the literature, PU/ND composites demonstrate comparable or superior mechanical, thermal, and shape-memory performance. Potential applications include smart actuators, flexible sensors, self-healing devices, and high-performance shape-memory components. The study is limited by rheological testing conducted only via frequency sweep below T_g_ and the narrow ND loading range (0.1–0.5 wt.%). Future work will focus on temperature-dependent rheology, higher filler concentrations, and exploring hybrid PU blends to further enhance multifunctional properties. Overall, the results highlight ND as a promising nanofiller for developing high-performance, multifunctional PU-based smart materials.

## Figures and Tables

**Table 1:** Summary of previous studies.

PU Chemical Structure	Filler Material	Property Evaluated	Important Outcomes	Reference
Not disclosed	Carbon black (CB) and MWCNTs	Mechanical & shape-memory properties	MWCNTs demonstrated improved shape-memory performance, increased the fixity ratio to 97% and boosted T_g_ by 10 °C.	[31]
Not disclosed	Carbon nanotube (CNTs)	Tensile test	The nanocomposite demonstrated a tensile strength of 15.4 MPa and an elongation at break of 420% Compared to pure PU.	[32]
Not disclosed	Carbon nanotubes (CNTs)	Tensile test	The tensile strength and elongation at break increased by 97% and 25%, respectively.	[33]
Not disclosed	Nano-silicon dioxide (SiO_2_)	Tensile & flexural test	Nano-SiO_2_ achieved notable increases in flexural strength (33.87%), tensile strength (20.75%), and interlaminar shear strength (66.54%) over neat	[34]
Not disclosed	Mini-sized graphene (mg)	Tensile test and thermal analysis	Improved tensile strength by 81%, modulus by 126.7%, and elongation by 605%, while also enhancing thermal conductivity by 17 times (4.24 W/m·K) compared to PU.	[35]
Not disclosed	Carbon nanotube (CNT) & graphene (G)	Tensile test	Tensile strength reaching 69.5 MPa and toughness 246.2 MJ/m^3^, corresponding to 1.9- and 2.9-fold increases, respectively, over pure TPU.	[36]
Not disclosed	Carbon black (CB)	Thermal and mechanical properties	Thermal stability increased from 220 °C to 270 °C with CB addition, and 5 wt.% CB demonstrated the highest mechanical qualities (8.02 MPa tensile strength, 434.25% elongation).	[37]
Soft segment: Poly (ε-caprolactone) diol (PCL-diol) and Poly(2-ethyl-2-oxazoline) diol (PEtOx-diol) Hard segment: Diisocyanate Chain extender: 1,4-butanediol (BDO)	None	Thermal properties (DSC, TGA). Dynamic mechanical analysis (DMA)	A melting peak near 50–60 °C (typical for crystalline PCL). The intensity decreased as PEtOx content increased.	[38]
Soft segment: Poly (1,6-hexanediol carbonate) diol (PCDL, M_n_; ≈ 2000 Da) Hard segment: 1,6-hexamethylene diisocyanate (HDI) Chain extender: 1,4-butanediol (BDO)	None	Thermal stability (TGA)	Decomposition onset ≈ 320–360 °C, major weight loss up to 430 °C.	[39]
Soft segment: Polycaprolactone (PCL) diol (M_n_ = 1000 g mol^−1^) Hard segment: Diphenylmethane diisocyanate-50 (MDI-50) chain extender: None	None	Shape-memory testing (thermo-responsive)	All bio-PUs showed excellent shape-memory performance. Best sample (PLA:PCL = 2:1) and noted a 98% recovery, full recovery in 15 s at 37 °C.	[40]
Soft segment: Poly (ε-caprolactone) diol (PCL, M_n_ ≈ 2000 g/mol) Hard segment: 4,4′-Diphenylmethane diisocyanate (MDI) Chain extender: 1,4-butanediol (BD)	None	TGA, DSC	Rotaxanes retarded recrystallization of PCL domains (less sharp melting peaks). No significant change in Tg across PU0–PU2.	[41]
Soft segment: Bio-based PHNA diols (polyhydroxynonanoate diols) Hard segment: 4,4′-Diphenylmethane diisocyanate (MDI) Chain extender: 1,4-Butanediol (BDO)	None	Shape-memory behavior	Shape-memory transition temperature (Ttrans): tunable between 32 and 51 °C, near body temperature.	[42]
Soft segment: Polyol (HO-R’-OH) Hard segment: Diisocyanate (OCN-R-NCO) Chain extender: HO-R’’-OH	None	Thermomechanical characterization, shape-memory behavior	Glass transition temperature (Tg) ≈ 65 °C. Shape fixity ratio: ~90%, Shape recovery ratio: ~93% at room temp.	[43]

**Table 2:** Shape fixity ratio (R_f_) and shape recovery ratio (R_r_) and at cycles 1, 2 and 3.

	Cycle 1		Cycle 2		Cycle 3		Cycle 1		Cycle 2		Cycle 3	
Samples	R_f_ (%)	Error (%)	R_f_ (%)	Error (%)	R_f_ (%)	Error (%)	R_r_ (%)	Error (%)	R_r_ (%)	Error (%)	R_r_ (%)	Error (%)
Pure PU	81	3	80	3	79	3	52	3	51	3	49	3
0.1 PU/ND	82	2	81	2	80	2	65	2	63	2	62	2
0.2 PU/ND	83	3	83	3	80	3	72	3	75	3	74	3
0.3 PU/ND	85	2	84	2	82	2	80	2	78	2	77	2
0.5 PU/ND	87	3	86	3	85	3	84	3	83	3	82	3

## Data Availability

The original contributions presented in this study are included in the article. Further inquiries can be directed to the corresponding author.

## Abbreviations

The following abbreviations are used in this manuscript:

PU	Polyurethane
ND	Nanodiamond
SMP	Shape-Memory Polymer
DSC	Differential Scanning Calorimetry
TGA	Thermogravimetric Analysis
XRD	X-ray Diffraction
SEM	Scanning Electron Microscope
TEM	Transmission Electron Microscopy
EDX	Energy Dispersive X-ray
SAED	Selected Area Electron Diffraction
COF	Coefficient of Friction
T_g_	Glass Transition Temperature
T_m_	Melting Temperature
T_onset_	Onset Temperature
R_f_	Shape Fixity Ratio
R_r_	Shape Recovery Ratio
G′	Storage Modulus
G′′	Loss Modulus
tan δ	Loss Factor (Damping Factor)
